# Supplementary material for: Structural defects in amyloid-β fibrils drive secondary nucleation
Source: Nat Commun. 2026 Feb 18;17:1933. doi: 10.1038/s41467-026-69377-1 (PMC12923811; doi:10.1038/s41467-026-69377-1)
Supplement: Supplementary file 1 — Supplementary Information [file 41467_2026_69377_MOESM1_ESM.pdf]

Supporting Information: Structural defects in amyloid-β fibrils drive secondary nucleation

Jing Hu[1,2], Tom Scheidt[3], Dev Thacker[2,4], Emil Axell[2], Elin Stemme[2], Urszula Łapińska[5], Stefan Wennmalm[6], Georg Meisl[3,7], Samo Curk[8], Maria Andreasen[9], Michele Vendruscolo[3], Paolo Arosio[10], Anđela Šarić[8], Jeremy D. Schmit[11], Tuomas P. J. Knowles[3,12], Emma Sparr[1], Sara Linse[2], Thomas C. T. Michaels[13,14], Alexander J. Dear[2,13,14*]

[1]Division of Physical Chemistry, Department of Chemistry, Lund University, 22100, Lund, Sweden.
[2*]Department of Biochemistry and Structural Biology, Lund University, 22100, Lund, Sweden.
[3]Centre for Misfolding Diseases, Yusuf Hamied Department of Chemistry, University of Cambridge, Lensfield Road, Cambridge, CB2 1EW, United Kingdom.
[4]Astbury Centre for Structural Molecular Biology, School of Molecular and Cellular Biology, Faculty of Biological Sciences, University of Leeds, Leeds, LS2 9JT, United Kingdom.
[5]Living Systems Institute, University of Exeter, Exeter, Devon, EX4 4QD, United Kingdom.
[6]Department of Applied Physics, Biophysics Group, SciLifeLab, Royal Institute of Technology-KTH, Solna, 171 65, Sweden.
[7]UK Dementia Research Institute, University of Cambridge, Hills Road, Cambridge, CB2 0AH, United Kingdom.
[8]Institute of Science and Technology Austria, Klosterneuburg 3400, Austria.
[9]Department of Biomedicine, Aarhus University, Aarhus, 8000, Denmark.
[10]Department of Chemistry and Applied Biosciences, Institute for Chemical and Bioengineering, ETH Zurich, Zurich, 8093, Switzerland.
[11]Department of Physics, Kansas State University, Manhattan, KS 66506, United States of America.

[12]Cavendish Laboratory, University of Cambridge, J J Thompson Avenue, Cambridge, CB3 0HE, United Kingdom.
[13]Institute of Biochemistry, Department of Biology, ETH Zurich, Zurich, 8093, Switzerland.
[14]Bringing Materials to Life Initiative, ETH Zurich, Zurich, Switzerland.

*Corresponding author(s). E-mail(s): alexander.dear@bc.biol.ethz.ch;

1 Supplementary Figures

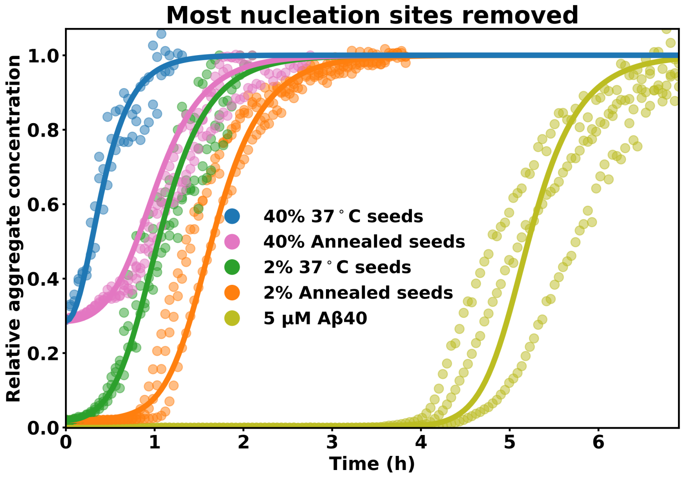

Supplementary Fig. 1 Aggregation of 5 μM Aβ40 at 37°Cwith 0%, 2% or 40% 37°C seeds or annealed seeds. The aggregation is monitored by ThT intensity. The fitting with the model of removing most nucleation sites is shown in lines.

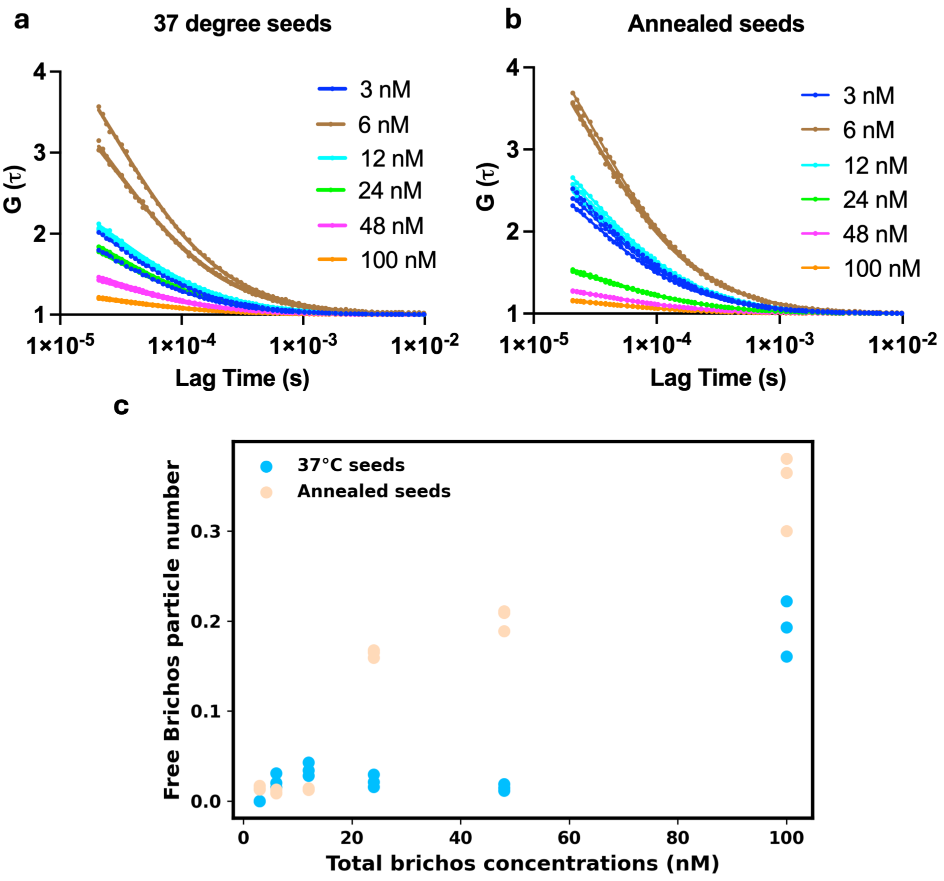

Supplementary Fig. 2 FCS experments of Aβ40 fibrils and Brichos. **a** and **b**: FCS fitting of the data for samples containing 4.5 μM 37°C fibrils or annealed fibrils, respectively. The fitting is done with 2 parameters fitting model while fixing the diffusion time of 27 μs (free dye) and 126 μs (Alexa488-Brichos). **c**: Free Brichos particle number gained from fitting in (a) and (b) is plotted against the total Brichos concentration.

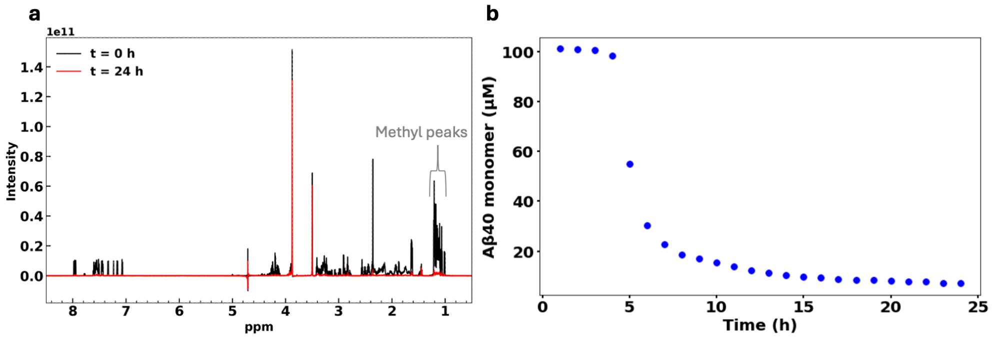

Supplementary Fig. 3 NMR experments of AβM1-40 at 60℃. **a**: The ^{1}H NMR spectra of 101 μM AβM1-40 at 60℃ at t=0 hour (black) or t=24 hours (red). **b**: AβM1-40 monomer concentration plotted against the time of incubation. The Aβ40 monomer concentration is calculated through integration of the methyl group peaks.

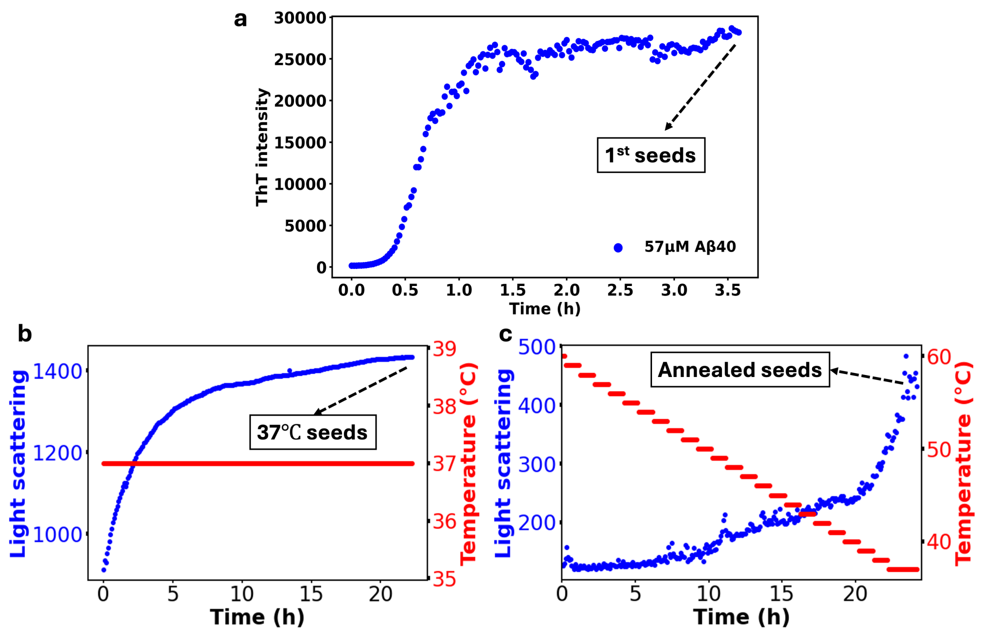

Supplementary Fig. 4 Generation of 37°C fibrils or annealed fibrils. **a**: The aggregation of 57 μM Aβ40 at 37°C is monitored by tracing the ThT (10 μM) intensity. A sample in the other well of the plate without ThT was incubated at the same time, and was taken at the end of aggregation as first seeds for generating 37°C fibrils or annealed fibrils. **b** and **c**: The aggregation of 10 μM Aβ40 with 2% first seeds at 37°C or 60 to 37°C is monitored by light scattering. The fibrils at the end of aggregation is collected as 37°C seeds or annealed seeds. Note that the light scattering values do not precisely quantify fibril mass, since sedimented fibrils are not detectable.

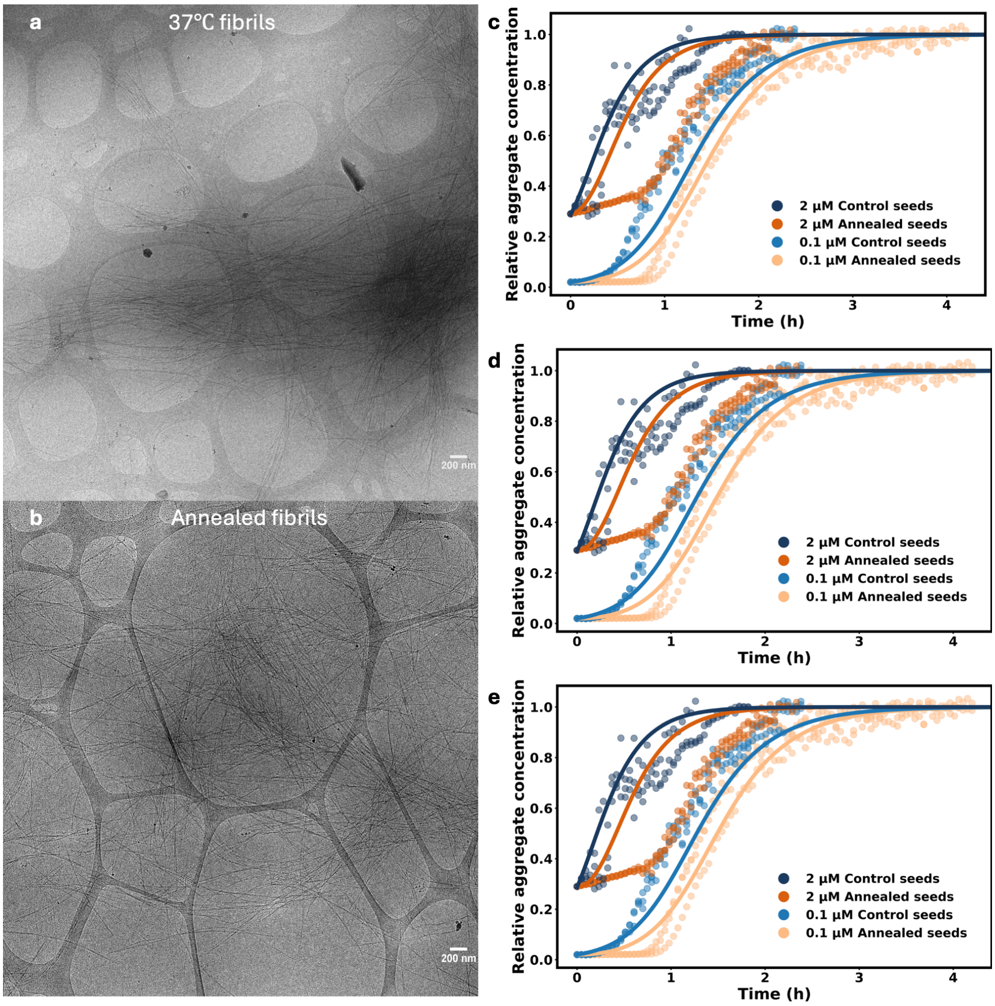

Supplementary Fig. 5 **a** and **b**: Cryo-TEM images of 37°C or annealed Aβ40 fibrils at low magnification. Typical lengths of 37°C and annealed fibrils appear similar, both in the µm range previously reported for non-annealed Aβ40 fibrils[1]. **c**, **d** and **e**: Misfits using kinetic models in which annealed and control fibril seeds have the same secondary nucleation propensity but average lengths that differ by factor of 10, 100 and 1000, respectively. These misfits are markedly worse than those presented in the main text using an unrealistically large length difference of 100 trillion-fold.

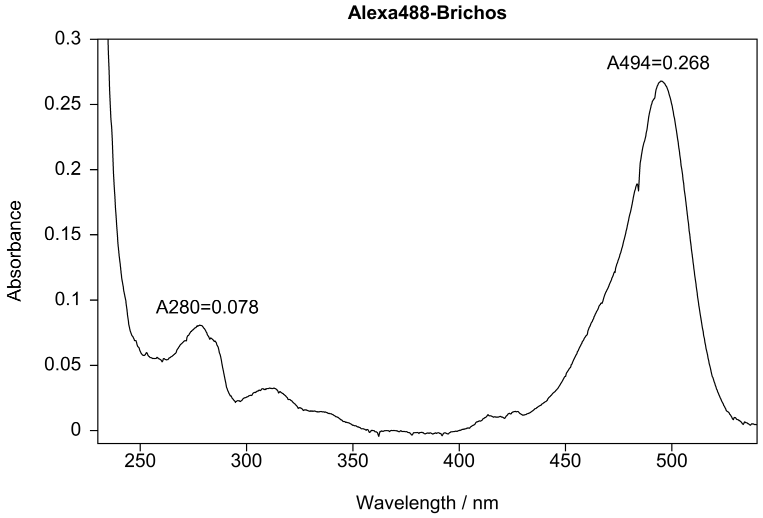

Supplementary Fig. 6 UV–vis absorbance spectrum of Alexa488–BRICHOS. The peak at 494 nm ($A_{494} = 0.268$) was used to determine the Alexa488 concentration. The measured $A_{280} = 0.078$ was corrected using correction factor $\mathrm{CF}_{280/494} = 0.11$ to obtain the protein concentration used to compute the degree of labelling.

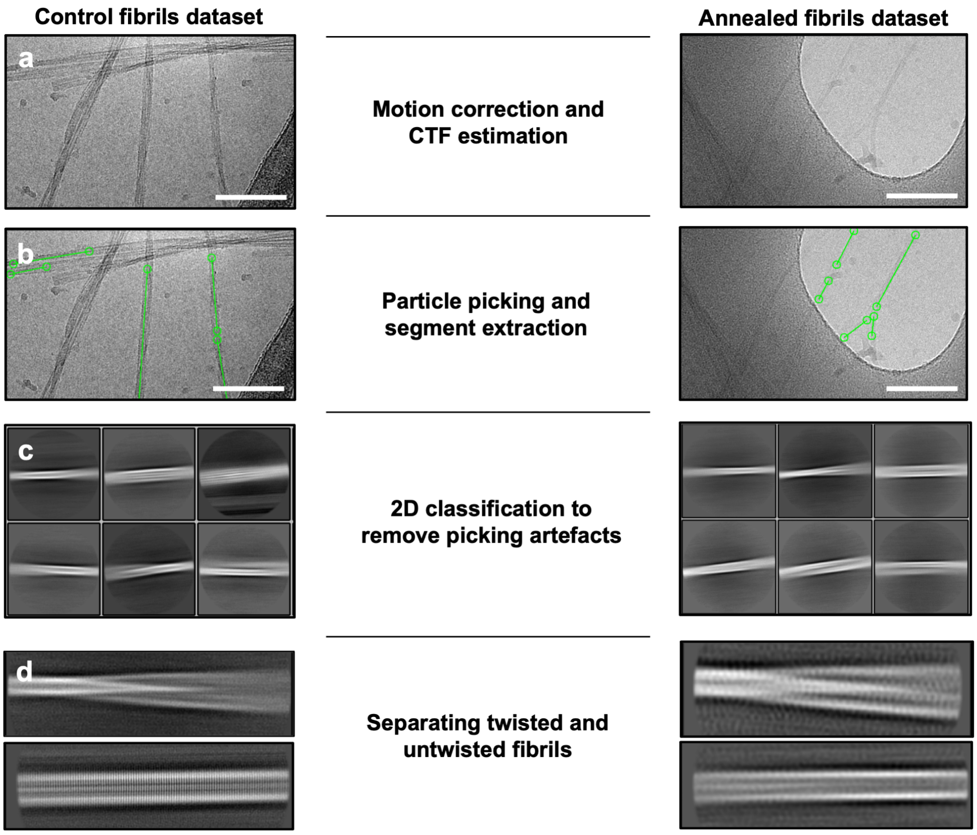

Supplementary Fig. 7 Cryo-EM data processing workflow for images shown in Fig.4 in the main text. The scale bars are 100 nm.

2 Supplementary Tables

Supplementary Table 1 Critical concentrations (solubility) of Aβ40 at different temperatures. The solubility data of 37 ℃ is measured by mass spectrometry and MDS [2], all other data points are measured by NMR.

**Temperature (°C)**	**Solubility (μM)**
27	1.19
37	0.36
45	4.84
50	6.18
60	7.27

Supplementary Table 2 Critical concentrations (solubility) of Aβ40 at different temperatures. The solubility data are adapted from [3]

**Temperature (°C)**	**Solubility (μM)**
19	8.14 ± 1.65[1]
28	1.72 ± 0.49
37	0.52 ± 0.05
46	1.62 ± 0.78
55	3.32 ± 0.66
64	15.44 ± 0.56

[1]Number of repeats per temperature = 6. The ± values are 95% confidence intervals calculated from those replicates.

Supplementary Table 3 Reported dissociation constants (K_D) of Brichos and amyloid monomers binding to fibril surfaces and secondary nucleation sites. *(Inferred from the complete saturation of secondary nucleation at 37°C above these monomer concentrations)

System	Binding partner	Site type	K_D	Conditions
Aβ42 fibrils [4]	Bri2 Brichos	Tight site	12.9 nM	37°C, pH 8.0
Aβ42 fibrils [4]	Bri2 Brichos	Loose site	18.5 μM	37°C, pH 8.0
Aβ42 fibrils [5]	proSP-C Brichos	Secondary nucleation site	40 nM	37°C, pH 8.0
Aβ40/42 fibrils [1, 6]	Aβ40/42 monomer	Secondary nucleation site	$\sim$ 6 μM*	37°C, pH 7.4/8.0
α-syn fibrils [7]	Bri2 Brichos	Tight site	22 nM	25°C, pH 7.4
α-syn fibrils [7]	Bri2 Brichos	Loose site	350 μM	25°C, pH 7.4
α-syn fibrils [7]	Bri2 Brichos	Averaged (1-site fit)	2.3 μM	25°C, pH 7.4
α-syn fibrils [7]	Bri2 Brichos	Secondary nucleation site	$\ll$ 1 μM	25°C, pH 7.4
α-syn fibrils [8]	Brichos variants	Averaged (1-site fit)	0.45–1.3 μM	21°C, pH 4.8
α-syn fibrils [9]	α-syn monomer	Loose site	$\sim$ 1 mM	pH 7
α-syn fibrils [10]	α-syn monomer	Tight site	$<$ 10 μM*	37°C, pH 5.5

3 Supplementary Methods

3.1 Equilibrium thermodynamics of fibril growth defects

Amyloid fibrils consist of stacked layers of small numbers of monomers, typically 2-5, typically with tens of thousands or millions of layers per fibril. Given the periodic symmetry along the long axis of (defect-free) fibrils, it is easiest to view the thermodynamics of their assembly layer-by-layer. Instead of considering discrete defect sites, we therefore consider defective layers, which may come in a variety of forms. They could contain one or more partially misfolded monomers (misfolded from the perspective of the regular amyloid structure). They could also be offset or misaligned compared to the preceding layer, but contain otherwise largely correct bonding within the layer. However, we do not need to make any assumptions as to the precise structure of the defective layer, other than that it is not so irregular as to prevent subsequent layers of monomers binding in the correct conformation for the regular structure. For the defect to become kinetically trapped, and located far from the fibril ends, this is in fact a requirement rather than an assumption.

3.1.1 Derivation of equilibrium defect stoichiometry formulae

We denote the free energy of forming a new layer as G_i, where $i = 0$ denotes a layer of the regular structure, $i = 1$ is the most-stable-possible defective layer that allows subsequent regular growth, $i = 2$ is the next-most-stable-possible defective layer, etc. At equilibrium, the probability for including a given growth defect in a fibril is then:

$$p_{eq,i} = \frac{e^{-G_i/RT}}{\sum_j e^{-G_j/RT}}. \tag{1}$$

If a defective layer of type i has n_i discrete defect sites, then the overall defect site stoichiometry is:

$$s_{eq} = \frac{1}{x}\sum_i n_i\, p_{eq,i}, \tag{2}$$

where x is the number of monomers in a layer. The sum can run from $i = 1$ or $i = 0$ since $n_0 = 0$.

Now, if defective layers are rare, which is equivalent to them being thermodynamically quite unstable compared to correctly-assembled layers (and quite likely *a priori*, given the highly regular nature of the amyloid structure), then the defect probability becomes:

$$p_{eq,i} \simeq e^{-\Delta G_i/RT}, \quad \Delta G_i = G_i - G_0. \tag{3}$$

Furthermore, if one type of defective layer is significantly more stable than others (or is a member of a class of defective layers of equal stoichiometry n_i and similar G_i values), then we can write:

$$p_{eq} = \sum_i p_{eq,i} \simeq p_{eq,1}, \tag{4}$$

and:

$$s_{eq} \simeq \frac{n_{\mathrm{def}}}{x} e^{-\Delta G_{\mathrm{def}}/RT}, \tag{5}$$

where we have for notational convenience defined $\Delta G_1 = \Delta G_{\text{def}}$ and $n_1 = n_{\text{def}}$. For instance, a lateral misalignment creates two defect sites, one on each side of the fibril. So, the equilibrium stoichiometry of defect sites per monomer is $s_{eq} = 2p_{eq}/x$.

Note, if the defective layer leads to weaker bonding both to the preceding and subsequently-assembled layers in the fibril, the bonding energy reduction in both planes must be included in G_i.

3.1.2 Calculation of defect free energies

The average annealed Aβ40 formation temperature is approximately the temperature at which half of the monomer has been aggregated. If protein aggregation were instantaneous, the solubility vs temperature chart (Supplementary Fig. 4**c**) would imply this to be around 45℃. The defect stoichiometry in annealed fibrils, $s = 1/834$, is therefore approximately s_{eq} at 45℃. This very low stoichiometry already implies that defects are unstable and that Eq. (3) therefore applies. Under the approximation that we ignore the contributions of all but the most stable type of defect, we can also use Eq. (5). The free energy penalty of formation of a defective layer is then

$$\Delta G_{\text{def}} = RT \ln \left(s_{eq} \frac{x}{n_{\text{def}}} \right). \tag{6}$$

We can now consider several cases.

4-monomer-thick fibril

For a misalignment defect and a 4-monomer-thick fibril, $x = 4$ and $n_1 = 2$ and the free energy penalty per layer is:

$$\Delta G_{\text{def}} = -16.0 \text{ kJ/mol}. \tag{7}$$

For a partially-misfolded single monomer defect, $n_1 = 1$ and the energy penalty is instead:

$$\Delta G_{\text{def}} = -14.1 \text{ kJ/mol}. \tag{8}$$

For comparison it is useful to have a value for G_0. This can be calculated from the solubility c_{eq}, which at 45℃ is 4.84 µM (see below):

$$c_{eq} = K_D = e^{(G_0/x)/RT}. \tag{9}$$

Doing the calculation gives:

$$G_0 = -129 \text{ kJ/mol}. \tag{10}$$

note the bonding free energy for a single correctly-bound monomer is -32.3 kJ/mol at this temperature.

So, if the defect is a misalignment defect, it incurs approximately a 1/8 energy penalty, or approximately 50% of the bonding energy of a single correctly-bound monomer. If it is a single-monomer defect, it is slightly less than this at ca. 43%. Either way, these defective layers are clearly thermodynamically very unstable.

2-monomer-thick fibril

For a misalignment defect and a 2-monomer-thick fibril, $x = 2$ and $n_1 = 2$ and the free energy penalty per layer is:

$$\Delta G_{\text{def}} = -17.8 \text{ kJ/mol.} \tag{11}$$

For a partially-misfolded single monomer defect, $n_1 = 1$ and the energy penalty is instead:

$$\Delta G_{\text{def}} = -16.0 \text{ kJ/mol.} \tag{12}$$

G_0 is now:

$$G_0 = -64.7 \text{ kJ/mol.} \tag{13}$$

These defective layer free energy penalties are slightly larger in absolute terms and much larger relative to the free energy for a correctly-bound layer.

Again, such defects are very thermodynamically unstable, with their free energy penalties being several multiples of $RT = 2.64$ kJ/mol.

3.2 Nonequilibrium growth defect stoichiometry

As derived above, when defects are the equilibrium dislocation defect stoichiometry is given by the exponential of the misalignment free energy penalty. This can be rewritten as:

$$p_{mis} = \frac{e^{-G_{\text{def}}/RT}}{e^{-G_0/RT}} = \frac{K_{\text{def}}}{K_0}, \tag{14}$$

where G_{def} is the free energy of defect formation and G_0 the free energy of normal or correct elongation, and K_{def} and K_0 the associated equilibrium constants.

For simplicity we consider a 1-dimensional fibril with one monomer per plane. Then the expression in terms of equilibrium constants can be expanded as:

$$p_{eq} = \frac{2k_{+,d}}{k_{\text{off},d}} \frac{k_{\text{off}}}{2k_+}. \tag{15}$$

Here, k_+ and k_{off} have the usual meanings of the elongation and depolymerization rate constants (for correctly bound monomers), and $k_{+,d}$ and $k_{\text{off},d}$ are the elongation and depolymerization rate constants for misaligned monomers.

In the limit of high supersaturation, essentially no depolymerization occurs and the stoichiometry of dislocations becomes just the ratio of elongation rates. Calling the stoichiometry in this "kinetic" limit p_{neq}, this is then $p_{neq} = k_{+,d}/k_+$. Our intuition is that increased kinetic trapping causes increased defect stoichiometry, i.e. $p_{neq} > p_{eq}$. This is true when:

$$\frac{k_{+,d}}{k_+} > \frac{2k_{+,d}}{k_{\text{off},d}} \frac{k_{\text{off}}}{2k_+}. \tag{16}$$

In turn, this is true if and only if:

$$\frac{k_{\text{off},d}}{k_{\text{off}}} > 1, \tag{17}$$

i.e. misaligned monomers detach more rapidly from fibril ends than correctly aligned monomers. This would seem to be self-evidently true, since they are not only less strongly bound but also closer in structure to soluble monomeric protein.

3.3 Solubility at different temperatures

The aggregation of AβM1-40 between 27 and 60 °C was monitored by integrating the methyl-group region of the AβM1-40 ^{1}H NMR spectra, recorded until a plateau was reached. For example, Supplementary Figure 3**a** shows spectra of Aβ M1-40 at 60 °C at time 0 and after 24 h, while Supplementary Figure 3**b** shows the methyl-group integration over time. Using this approach, the solubility of Aβ M1-40 at different temperatures was determined (Tables 1). At 37 °C, the solubility was too low to yield measurable NMR signals, and was therefore not determined by NMR. However, previous studies from our laboratory have measured solubility at 37 °C by quantifying peptide concentration in the supernatant using MDS and MS. We also compared our results with Aβ1-40 solubility data reported by another group [3] (Tables 2). Both datasets show a consistent trend of increasing solubility with either increasing or decreasing temperature relative to 37 °C. Differences between the solubilities reported in Tables 1 and 2 may arise from several factors: (i) monomer preparation: in our study, freshly purified Aβmonomers were used directly, whereas in their protocol lyophilized peptide was subjected to multiple chemical treatments (TFA and HFIP dissolution, evaporation, freeze–thaw cycles, and re-dissolution in NaOH) before experiments, (ii) the measurement method (direct NMR integration vs. centrifugation followed by HPLC), (iii) temperature control (constant set temperature during our NMR experiments vs. centrifugation at 4 °C following incubation at the target temperature).

3.4 Static light scattering

The aggregation reaction under changing temperature was monitored in quartz cuvettes using a Probe Drum instrument (Probation Labs) by recording static light scattering at 5-minute intervals over up to 24 hours, as shown in Supplementary Figure 4. The static light scattering signal was obtained by measuring the intensity of the excitation red laser at a 90° angle to the incident path. The 37℃ seeds were generated by incubating 10 µM Aβ40 with 2% seeds at 37℃ in the Probe Drum for at least 24 hours. The annealed seeds were produced by incubating 10 µM Aβ40 with 2% seeds, gradually cooling from 60℃ to 37℃ at a rate of 1℃ per hour, and then storing them at 37℃ until further experiments.

3.5 Cryo-TEM imaging for fibril length differences

Cryo-EM imaging of 37℃ or annealed Aβ40 fibrils were performed as described previously [11]. As shown in Supplementary Figure 5, the fibrils are entangled, making it difficult to determine the precise length of individual fibrils. Nevertheless, the annealed fibrils do not appear noticeably shorter than those formed at 37℃. Samples were vitrified on glow-discharged lacey carbon grids (300 mesh) using a Leica EM GP plunge freezer at 20℃ and 90% relative humidity, and frozen in liquid ethane at –184℃.

Grids were transferred to a JEM 2200FS electron microscope using a Fischione 2550 cryo-holder, and imaged at 200 kV under low-dose conditions with a 10 eV energy filter. Zero-loss images were acquired using a TVIPS F416 camera and processed using SerialEM and ImageJ.

3.6 Alexa-488 labelling efficiency on BRICHOS

We quantified the degree of labelling of Alexa Fluor 488 on Brichos by UV–visible absorbance, as shown in Supplementary Figure 6. The dye concentration was derived from the absorbance at 494 nm, while the protein concentration was obtained from the dye-corrected absorbance at 280 nm, as shown below:

$$\begin{aligned}
C_{\text{dye}} &= \frac{A_{494}}{\varepsilon_{494}^{\text{Alexa488}}\, l} \\
A_{280,\text{dye}} &= \text{CF}_{280/494}\, A_{494} \\
C_{\text{protein}} &= \frac{A_{280} - A_{280,\text{dye}}}{\varepsilon_{280}^{\text{BRICHOS}}\, l} \\
\text{degree of labelling} &= \frac{C_{\text{dye}}}{C_{\text{protein}}}
\end{aligned}$$

$l = 1$ cm, $\varepsilon_{494}^{\text{Alexa488}} = 72{,}000\ \text{M}^{-1}\text{cm}^{-1}$, $\varepsilon_{280}^{\text{BRICHOS}} = 11{,}500\ \text{M}^{-1}\text{cm}^{-1}$, $\text{CF}_{280/494} = 0.11$, $A_{494} = 0.268$, $A_{280} = 0.078 \Rightarrow C_{\text{dye}} = 3.72\ \mu\text{M}$, $A_{280,\text{dye}} = 0.0295$, $C_{\text{protein}} = 4.22\ \mu\text{M}$, **degree of labelling** $= 0.90$.

4 Supplementary Discussion

In the main text Discussion we proposed that fragmentation may occur preferentially at weaker growth defects. This hypothetical fragmentation mechanism could be explicitly tested by comparing the length distributions of annealed and non-annealed fibrils after gentle sonication using microscopy. Although this technique is unsuitable for Aβ40 fibrils, which are too strong to fragment significantly even under relatively aggressive sonication [12], it could be applied to weaker fibrils such as prions provided an annealing protocol is developed that does not affect their morphology. Conversely, a decrease in observed length distribution after annealing and sonication could be used as additional evidence of a successful annealing procedure. Note, however, detecting truly representative differences in length distributions can be challenging using microscopy, which is heavily biased towards the shorter, better-dispersed fraction of fibrils, with the majority of larger fibrils often remaining in large, un-analyzable clumps [12].

Assuming this mechanism holds true, fragmentation is nonetheless extremely unlikely to remove appreciable fractions of defects in amyloid fibrils that have not been strongly sonicated. Otherwise, the average fibril length would be comparable to the spacing between the original defect sites. Since fibrils are typically micron-scale and consist of tens of thousands to tens of millions of monomers, this is clearly impossible unless defects are negligibly rare compared to those we have found in Aβ. This in turn would require the fibrils to be far more thermodynamically stable than Aβ fibrils. *In situ* breakage and repair of single-filament defects in a multi-filament fibril is also unlikely to be a significant defect sink. This follows since the non-defective filaments would not only protect the defective filament from fragmentation but also hold the defective plane(s) in the incorrect geometry even after its breakage.

Supplementary References

[1] Meisl, G., Yang, X., Hellstrand, E., Frohm, B., Kirkegaard, J.B., Cohen, S.I.A., Dobson, C.M., Linse, S., Knowles, T.P.J.: Differences in nucleation behavior underlie the contrasting aggregation kinetics of the Aβ40 and Aβ42 peptides. Proc. Natl. Acad. Sci. U.S.A. **111**, 9384–9389 (2014) https://doi.org/10.1073/pnas.1401564111

[2] Lattanzi, V., Bernfur, K., Sparr, E., Olsson, U., Linse, S.: Solubility of aβ40 peptide. JCIS Open **4**, 100024 (2021)

[3] Hicks, D.E.: Thermophysical properties of the Amyloid Beta protein from differential scanning calorimetry (2005). Master's thesis, University of Tennessee, Knoxville, Department of Chemical Engineering, Aug 2005. https://trace.tennessee.edu/utk_gradthes/2694

[4] Kumar, R., Le Marchand, T., Adam, L., Bobrovs, R., Chen, G., Fridmanis, J., Kronqvist, N., Biverstål, H., Jaudzems, K., Johansson, J., Pintacuda, G., Abelein, A.: Identification of potential aggregation hotspots on aβ42 fibrils blocked by the anti-amyloid chaperone-like brichos domain. Nature Communications **15**(1), 965 (2024) https://doi.org/10.1038/s41467-024-45192-4

[5] Cohen, S.I.A., Arosio, P., Presto, J., Kurudenkandy, F.R., Biverstal, H., Dolfe, L., Dunning, C., Yang, X., Frohm, B., Vendruscolo, M., Johansson, J., Dobson, C.M., Fisahn, A., Knowles, T.P.J., Linse, S.: A molecular chaperone breaks the catalytic cycle that generates toxic ab oligomers. Nat. Struct. Mol. Biol. **22**, 207–213 (2015)

[6] Cohen, S.I., Linse, S., Luheshi, L.M., Hellstrand, E., White, D.A., Rajah, L., Otzen, D.E., Vendruscolo, M., Dobson, C.M., Knowles, T.P.: Proliferation of amyloid-β42 aggregates occurs through a secondary nucleation mechanism. Proceedings of the National Academy of Sciences **110**(24), 9758–9763 (2013)

[7] Adam, L., Kumar, R., Arroyo-Garcia, L.E., Molenkamp, W.H., Nowak, J.S., Klute, H., Farzadfard, A., Alkenayeh, R., Nielsen, J., Biverstål, H., Otzen, D.E., Johansson, J., Abelein, A.: Specific inhibition of α-synuclein oligomer generation and toxicity by the chaperone domain bri2 brichos. Protein Science **33**(8), 5091 (2024) https://doi.org/10.1002/pro.5091 https://onlinelibrary.wiley.com/doi/pdf/10.1002/pro.5091

[8] Ghosh, D., Torres, F., Schneider, M.M., Ashkinadze, D., Kadavath, H., Fleischmann, Y., Mergenthal, S., Güntert, P., Krainer, G., Andrzejewska, E.A., Lin, L., Wei, J., Klotzsch, E., Knowles, T., Riek, R.: The inhibitory action of the chaperone brichos against the α-synuclein secondary nucleation pathway. Nature Communications **15**(1), 10038 (2024) https://doi.org/10.1038/s41467-024-54212-2

[9] Kumari, P., Ghosh, D., Vanas, A., Fleischmann, Y., Wiegand, T.,

Jeschke, G., Riek, R., Eichmann, C.: Structural insights into α-synuclein monomer–fibril interactions. Proceedings of the National Academy of Sciences **118**(10), 2012171118 (2021) https://doi.org/10.1073/pnas.2012171118 https://www.pnas.org/doi/pdf/10.1073/pnas.2012171118

[10] Gaspar, R., Meisl, G., Buell, A.K., Young, L., Kaminski, C.F., Knowles, T.P.J., Sparr, E., Linse, S.: Secondary nucleation of monomers on fibril surface dominates α-synuclein aggregation and provides autocatalytic amyloid amplification. Quarterly Reviews of Biophysics **50**, 6 (2017) https://doi.org/10.1017/S0033583516000172

[11] Hu, J., Linse, S., Sparr, E.: Ganglioside micelles affect amyloid β aggregation by coassembly. ACS Chemical Neuroscience **14**(24), 4335–4343 (2023)

[12] Dear, A.J., Meisl, G., Hu, J., Knowles, T.P.J., Linse, S.: Kinetics of seeded protein aggregation: Theory and application. J. Chem. Phys. **163**(4), 045101 (2025) https://doi.org/10.1063/5.0273677
